# Personalized versus fixed tactile cueing in Parkinson’s disease: Protocol for a randomized controlled trial on gait automaticity

**DOI:** 10.1371/journal.pone.0336859

**Published:** 2025-11-21

**Authors:** Pablo I. Burgos, William Liu, Carla Silva-Batista, Francesca Baker-Alcalá, Patricia Carlson-Kuhta, Laurie A. King, Fay B. Horak, Kathryn A. Chung, Jodi A. Lapidus, Martina Mancini

**Affiliations:** 1 Department of Neurology, Oregon Health & Science University, Portland, Oregon, United States of America; 2 Doctor of Physical Therapy Program, George Fox University, Newberg, Oregon, United States of America; 3 School of Public Health, Oregon Health & Science University – Portland State University, Portland, Oregon, United States of America; PLoS ONE, UNITED STATES OF AMERICA

## Abstract

**Background:**

Gait automaticity, the ability of the brain to control locomotion with minimal use of executive-attentional resources, is altered in people with Parkinson’s disease (PD). Recently, we showed that step-synchronized tactile cueing improved gait regularity and freezing of gait in PD; however, it is not known if this cueing mode also improves gait automaticity. Thus, this study investigates the effects of step-synchronized tactile cueing (versus fixed cueing) on gait automaticity in the laboratory and during daily life.

**Methods:**

This is a pilot, randomized, double-blinded study where sixty participants with PD will be randomized into one of two, cueing interventions: 1) personalized, step-synchronized tactile cueing and 2) tactile cueing at fixed intervals. Both cueing interventions use vibrotactile stimulation of wrist bands. During a laboratory study visit, we will measure cortical activity with a wireless, portable functional near-infrared spectroscopy system (fNIRS) during walking tasks. Gait will be assessed using inertial sensors placed on the limbs and trunk. In addition, in daily life, participants will use the same cueing mode at home. The primary outcomes include prefrontal & primary sensory cortex activity. Secondary outcomes are gait stride time, gait local dynamic stability, turn duration and trunk jerk during turning as metrics of gait automaticity in the laboratory. Daily life gait and turning are exploratory measures.

**Discussion:**

This project will advance the understanding of brain mechanisms associated with walking automaticity during tactile cueing and provide the basis for innovative, personalized cueing to rehabilitate gait automaticity in people with PD.

**Trial registration:**

ClinicalTrials.gov NCT05818189.

## 1. Background

Gait automaticity, defined as walking with minimal cognitive effort [1], is a hallmark of healthy gait and it is typically governed by a distributed network, including the spinal cord, brainstem, cerebellum, basal ganglia and sensorimotor cortical areas [2–4]. In normal conditions, cognitive control regions (i.e., prefrontal cortex) remain minimally active unless the task or environment is novel or highly challenging [5].

In Parkinson’s disease (PD), gait abnormalities are often linked to a shift from brain automaticity to compensatory executive control [6]. This shift places increased demands on limited cognitive resources, and may negatively impact functional walking performance [7]. Impaired basal ganglia function likely contributes to this loss of automaticity [1].

To better understand gait automaticity, both neural and behavioral correlates must be studied [8–10]. While direct access to subcortical activity during gait is limited, cortical activity can be captured using EEG or functional near-infrared spectroscopy (fNIRS) [11,12]. Behaviorally, gait and turning quality can be measured objectively [13] and further evaluated under dual-task conditions to increase cognitive load [14].

EEG studies report increased activity in the prefrontal cortex (PFC), supplementary motor area (SMA), motor cortex, and sensorimotor cortex while walking in people with PD compared to healthy controls [15,16]. Such an overall increase in cortical activity while walking is consistent with functional magnetic resonance imaging (fMRI) studies while participants were lying in the scanner and operating a foot pedal mimicking walking [8]. fNIRS studies also report elevated PFC activity in PD during single-task walking, though findings during dual-task conditions are mixed [17,18]. Methodological inconsistencies, such as variability in signal processing, have limited generalizability across studies [11,19]. While the PFC is the most studied region, a few fNIRS studies have also explored other cortical areas during walking in people with PD [11,20].

Behaviorally, increased variability in gait (stride time, stride length, gait speed variability) [8,21] and impaired turning (velocity, smoothness) [22,23] have been associated with reduced automaticity. In healthy older adults, such variability remains stable during dual-task walking, whereas in PD, variability increases, suggesting increased reliance on cognitive control [24,25]. Turning appears particularly sensitive to automaticity loss [22]. However, gait and turning variability can also be influenced by freezing of gait (FOG) and postural instability, complicating interpretation [21,26]. Therefore, increased variability during gait and turning may not necessarily represent an automaticity decrement, so measuring cortical activity directly while walking would help validate automaticity changes [10]. Additionally, other studies show that very low and very high gait variability (U-shaped trend) could be associated with subclinical basal ganglia damage [27]. Thus, combining cortical and behavioral measures together may offer a more complete understanding of gait automaticity in PD, critical to developing improved rehabilitation strategies.

Current treatments for PD, such as levodopa or deep brain stimulation, do not reliably improve automaticity in PD. Levodopa does not improve, and may worsen, balance and attention [28,29], while deep brain stimulation offers limited benefits for balance or cognitive function [30]. As a result, alternative and complimentary interventions are needed.

Cueing, using external temporal or spatial stimuli like auditory, visual, or tactile stimuli [31], can improve gait, turning, and FOG [32]. Its effects vary depending on the cueing modality, medication state, and individual impairments [32]. However, the neural mechanisms underlying cueing remain unclear, particularly its impact on cortical areas like the PFC [33–36]. If cueing with external stimuli while walking requires increased cortical attention, we will see increased PFC activity, whereas if cueing improves automaticity, we will see decreased PFC activity. Thus, it is necessary to better understand the impact of different cueing modalities on the cortical and behavioral correlates of automaticity, to improve the effectiveness of cueing [37].

This study aims to evaluate personalized tactile cueing, synchronized to an individuals’ gait rhythm [38], on gait automaticity. The current use of cueing in rehabilitation predominantly uses cues in a fixed, open-loop manner (i.e., constant rhythmical sounds or vibrations, or visual cues as tape on the floor) rather than a personalized, closed-loop manner of cueing (i.e., intermittent stimuli set to the individual’s personal walking pace) [38]. This personalized mode of cueing has demonstrated a higher degree of gait and posture improvement and longer lasting effects compared with open-loop cueing [38–40]. Tactile cues may also engage more subcortical (automatic) pathways than visual or auditory cues [38], supporting proprioception and somatosensory integration, functions often impaired in PD [41–44].

Our previous work [38,40] showed that personalized, tactile cueing reduces FOG during turning in people with PD and improved gait stability, measured by local dynamic stability, during walking in people with PD. These effects may stem from enhanced proprioceptive feedback via cerebellar pathways. Early findings also suggest improvements in gait asymmetry and rhythmicity with peripheral tactile stimulation [45].

This study aims to test the feasibility and impact of using tactile cueing on gait in daily life. Since motor learning and gait automaticity depend on repetition [46], practicing cueing during everyday mobility may lead to longer-term improvements than cueing limited to clinic or laboratory sessions. Although several studies have trialed home-based cueing [31,47,48], they lacked objective gait and turning metrics in daily living, limiting insights into effects of cueing on gait regularity.

## 2. Objectives

The primary objective of this trial is to assess immediate changes in cortical activity during walking with two types of tactile cueing in people with PD, and whether these changes are accompanied by improvement in gait and turning (laboratory testing). We hypothesize that personalized tactile cueing will reduce PFC activity (improve automaticity) and reduce gait variability (as well as improve gait and turning speed). We also expect to observe increased activity in the primary sensory cortex with this type of personalized cueing that may require sensorimotor planning. In contrast, we predict that the fixed cueing will increase PFC activity and increase gait variability (need for more attention to the cues). An exploratory objective is to evaluate the feasibility and potential benefits of using tactile cueing during daily life.

## 3. Methods/Design

### 3.1. Study design and setting

**Fig 1:**
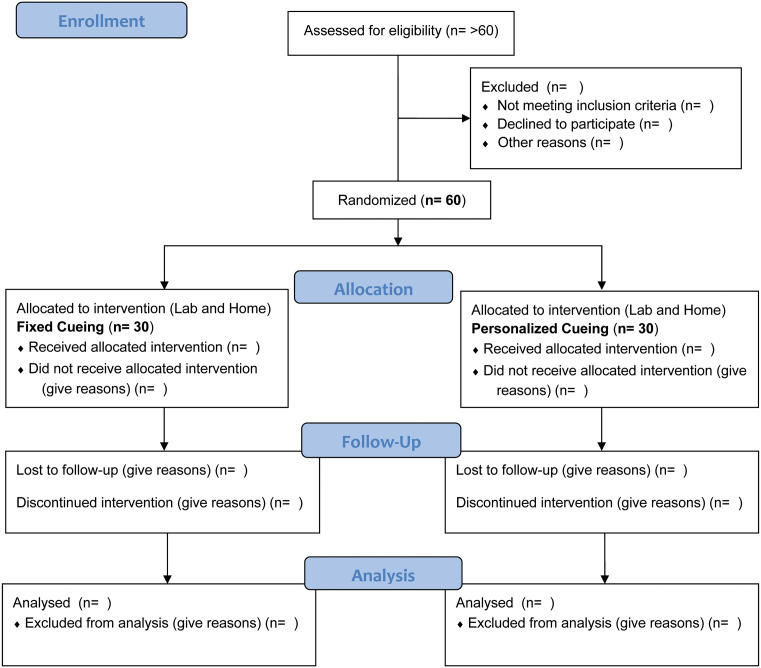
CONSORT flow diagram.

**Fig 2:**
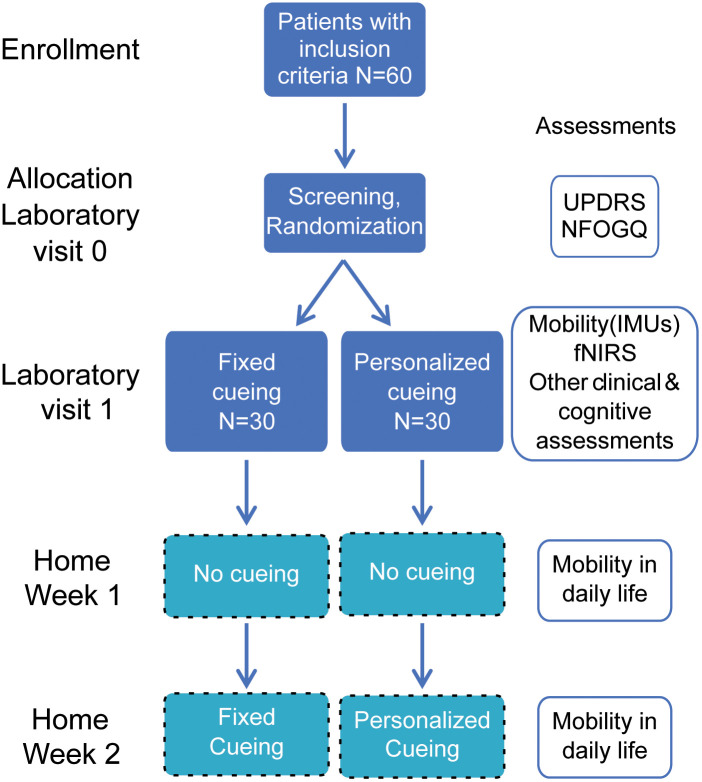
Activities and assessments during the clinical trial. See the section “Clinical and cognitive assessments” for the specific list of scales used.

This pilot randomized controlled trial (RCT) (NCT05818189) uses a parallel, double-blinded (investigators and participants), single-center design, to compare the effects of personalized tactile cueing versus fixed tactile cueing. The participants will not know which intervention is favored for improving gait automaticity, and the researchers collecting and analyzing the data will not know which intervention group participants were randomly assigned. Only the study coordinator and the laboratory engineer will know the intervention after the randomization to coordinate the visits and home intervention (see Figs 1 and 2). The allocation ratio will be 1:1, and the unit of randomization will be each participant.

A two-phase screening procedure will be conducted to determine participant eligibility. During Laboratory Visit 0, researchers will administer a clinical assessment and brief questionnaires in person to confirm inclusion criteria. Eligible participants will then be randomized via the REDCap randomization module, programmed by a blinded biostatistician, and assigned to either the personalized or fixed tactile cueing group.

At Laboratory Visit 1, a separate blinded researcher will conduct the baseline mobility assessment, which includes walking and turning trials using fNIRS and three wearable sensors (placed on the feet and lumbar region), as well as additional clinical and cognitive tests. Following this, the blinded researcher will leave the room and an unblinded coordinator and engineer will enter the lab to explain and configure the assigned cueing modality (approximately 15 minutes). The blinded researcher will then return to administer the same mobility protocol with cueing in place.

After completion of the laboratory assessment, a research team member will instruct the participants how to use, wear, and charge the cueing system (either fixed or personalized) and the wearable sensors for mobility monitoring and cueing at home. Participants will wear two instrumented socks and a lumbar sensor continuously for two weeks. They will be instructed to use the cueing system only during the second week of home monitoring.

To promote participant retention through all the study components, we will maintain regular contact with participants through a dedicated study coordinator who will be available to address any questions or concerns. We will also provide flexible scheduling for the visits and send appointment reminders via text or email. To compensate for their time and effort, participants will receive a monetary incentive at the completion of each visit and after each week of home participation.

Screening, intervention, and assessment visits will take place at the Balance Disorders Laboratory at Oregon Health & Science University (OHSU) in Portland, Oregon, USA. Daily life gait monitoring and cueing use will occur in participants’ homes.

### 3.2. Recruitment, screening and consent

Recruitment will be conducted via an existing database of people with PD, who have given informed consent to save and use their contact information for study participation, by referral of movement disorders specialists in the Portland metro area, and through community outreach events. Potential participants will be informed about study procedures and goals. If interested, inclusion and exclusion criteria will be evaluated by phone screening and Laboratory Visit 0. For people who wish to participate, an informed consent will be presented and explained verbally to the participants. The informed consent includes details on the purpose of the study, what the participants will do, possible risks and benefits of the study (without assigning superiority to any intervention). The consent also informs the participants that they may withdraw from the study at any time without penalty. Participants will be given ample opportunity to read, ask questions, and when satisfied, document their consent to participate by signing the informed consent form (written).

The start and end of the recruitment period are 1/15/2025 and 10/01/27 respectively.

*Inclusion criteria:* People with idiopathic PD, age range 55–85 years old, without musculoskeletal or peripheral or central nervous system disorders (other than PD) that could significantly affect their balance and gait. All participants will be capable of following directions for the protocols and giving informed consent. The UK Brain Bank criteria will be used for idiopathic PD diagnosis; i.e., bradykinesia and at least one of the following: rest tremor (4–6 Hz), muscular rigidity, and postural instability not caused by visual, vestibular, cerebellar, or proprioceptive dysfunction. Three or more of the following must be present for the diagnosis of idiopathic PD: unilateral onset, rest tremor, progressive, persistent asymmetry, and responsiveness to levodopa [49]. All PD participants will be Hoehn & Yahr Levels II-III.

*Exclusion criteria:* 1) Parkinson plus syndromes such as progressive supranuclear palsy, multiple system atrophy, corticobasal syndrome, possible vascular parkinsonism, 2) implanted electrodes for deep brain stimulation (DBS), 3) current use of dopamine-blocking agents or cholinesterase inhibitor (as may affect PFC activity while walking), 4) severe dyskinesia that may affect the quality of fNIRS, 5) major musculoskeletal or neurological disorders, structural brain disease, epilepsy, acute illness or health history, other than PD, significantly affecting gait and turning, i.e., peripheral neuropathy with proprioceptive deficits, musculoskeletal disorders, vestibular problem, head injury, stroke, 6) MoCA < 21 or dementia that precludes consent to participate or ability to follow testing procedures [50] and 7) Inability to stand or walk for 2 minutes without an assistive device.

Full ethical approval has been granted by the Oregon Health & Science university IRB (24837), in accordance with the principles of the Declaration of Helsinki.

### 3.3. Randomization and blinding

At the end of Laboratory Visit 0, participants will be randomized into either the personalized or fixed tactile cueing based on computer generated blocks with randomly permuted block size (2–6). We will use a stratified randomization to balance the disease severity per group, using Hoehn and Yahr (2 or 3) and FoG status (yes or no) (New Freezing of Gait Questionnaire (NFOGQ) Q1) [51]. Randomization procedures are fully concealed, and group allocation is conducted automatically within REDCap (a blinded statistician generated the stratification matrix). The information is only known by the study coordinator and the engineer, who are not participating in the data collection and data analysis. Different levels of access to REDCap are set for the study staff to prevent unblinding.

### 3.4. Assessment procedures

#### 3.4.1. General procedures.

Fig 2 below illustrates the flow of study activities. After screening and randomization, the immediate effect of the cueing interventions will be assessed during a study visit to the laboratory (Visit 1) through measures of cortical activity and functional mobility. Additionally, clinical and cognitive scales will also be conducted during this visit.

The main outcomes will be measured in the ON-medication state (after approximately 1-hour from last intake) before and while using their assigned tactile cueing mode (personalized or fixed). During the assessment, participants will complete the following mobility tasks, first without cueing and then with cueing activated: 1) 2-minute walking, 2) 2-minute walking with a concurrent cognitive dual-task, 3) 1-minute 360 degrees turning in place, and 4) 1-minute 360 degrees turning in place with a concurrent cognitive dual-task.

The gait task consists of two minutes of walking back and forth along a 9-meter hallway at comfortable speed. The turning task involves alternating 360° turns (right and left) in place for one minute. The secondary cognitive task used in both dual-task conditions is a modified version of the Continuous Performance Test–AX (AX-CPT), which assesses attention and vigilance.

Throughout the mobility protocol, participants will wear a portable fNIRS system (Brite and OctaMon, Artinis) and three wireless inertial sensors (APDM Precision Motion, Clario) positioned on the posterior trunk and both feet to objectively measure cortical activity while performing gait and turning tasks.

#### 3.4.2. Clinical and cognitive assessments.

The clinical assessment (additional to MDS-UPDRS and NFOGQ used in the randomization) includes the PDQ-39: Parkinson’s Disease Quality of Life questionnaire, which has 39 questions reflecting 8 domains of quality of life. Subscale scores and a summary index representing the global health-related quality of life will be calculated. The PDQ-39 reflects limitations to participation in community mobility [52]. In addition, the Apathy Scale (AS) consisting of 14 items phrased to be answered on a four-point Likert scale [53] and the Geriatric Depression Scale (GDS) [54]; both recommended for use in PD [55,56], will be used as covariates in all analyses as apathy and depression may influence gait and turning [57]. The cognitive assessment includes *Executive function and* will be measured using Royall’s clock drawing (**CLOX 1**) and Trail-making (**TMT**) Part A and B [58]. Impaired executive function will use the cut-off of 2.5 on the Trail-making B/A [59,60]. *Global cognition* will be assessed with the Montreal Cognitive Assessment (**MoCA**) [61]. *Attention* will be measured with a computerized button pressing battery, involving simple (**SRT**) and choice reaction time (**CRT**) [62]. *Working memory* will be measured through seated forward digit span and *visuospatial ability* with a judgement of line orientation (**JLO**) tasks and Royall’s **CLOX 2** tasks.

#### 3.4.3. Cortical activity assessment.

A portable fNIRS system (Artinis Medical Systems) will be used to record cortical activity at 50 Hz. A neoprene head cap (fiber holder) marked with labels of the international 10–20 electroencephalography system and predetermined locations for the optodes will be placed on the participant’s head. Specifically, a 36-channel (26 + 10) arrangement with 6 short channels, consisting of 18 transmitters and 10 detectors/receivers, will cover PFC, SMA, primary motor, primary sensory and primary visual cortices bilaterally. Six short-separation channels will record superficial extracerebral hemodynamic response. *fNIRS* outcome measures while walking and turning (bilateral PFC, SMA, parietal and occipital cortical activity). Relative changes in the concentrations of HbO_2_ while walking or turning tasks will be compared to baseline conditions (e.g., standing); similar to our previous publications [36,63–65]. Briefly, the OxySoft (v3.7.2, Artinis Medical Systems) software preprocesses the NIRS signal by converting raw light intensity values to oxy-hemoglobin (HbO_2_), deoxy-hemoglobin (HHb), and total hemoglobin concentrations [66]. To eliminate equipment noise, respiration and heart pulsation, a low-pass filter with a cut-off frequency of 0.14 Hz will be applied to the fNIRS signal [67]. HbO_2_ and HHb concentrations will be calculated as the average of the channels from both sides of the skull that are located over specific cortical regions (PFC, SMA, primary sensory, and visual), which will be defined using 3D digitization. Only changes in HbO_2_ will be considered for further analysis, as HbO_2_ has been shown to be the more reliable and sensitive measurement than HHB [67]. Short-separation channel data will be regressed from the HbO_2_ signal to discount superficial extra-cerebral signal interference [36,63–65].

#### 3.4.4. Gait and turning assessment (Laboratory and Daily life).

Gait and turning measures are reported in Table 1 and calculated using a combination of proprietary algorithms (Mobility Lab V2 system, APDM Wearable Technologies, a Clario company) and custom algorithms validated by our Balance Disorders Laboratory [13,68,69].

**Table 1:** Primary and secondary outcomes.

Cortical activity (HbO_2_)	Gait measures	Turning measures
**Prefrontal**	Stride velocity (cm/sec) and CoV	# Steps to complete a turn
**Primary sensory**	Stride length (m) and CoV	Turn peak velocity (degree/s)
Supplementary Motor Area and primary motor	Stride time (s) and CoV	Turn duration (sec)
Primary visual	Local Dynamic Stability (LDS,-)	Turn trunk jerk (m^2^/sec^2^)

**Primary outcome in bold.**

In the laboratory, during walking trials, stride time variability (a key marker of automaticity) will be calculated as the coefficient of variation of stride time. Phase-dependent local dynamic stability (LDS) will be estimated using methods previously described in [40]. For the 360° turning in place task, we will analyze the yaw angular velocity from the posterior trunk sensor to compute [70] the average turning duration, number of steps in turn, and the average jerkiness of the turns.

#### 3.4.5. Daily Life mobility monitoring.

During the two-week home monitoring period, participants will wear two instrumented socks (one per foot) and a lumbar Opal sensor, secured with an elastic belt. These devices are to be worn for at least 8 hours/day during daily activities. The socks and lumbar sensor contain the same inertial measurement units as the standard Opals, including a tri-axial accelerometer, gyroscope, and magnetometer, sampling at 128 Hz. The sock design separates the battery (placed above the lateral malleolus) from the sensor (mounted on the foot dorsum), as detailed in [23,71–74]. The sensors are lightweight (22 g), store up to 30 days of data, and offer a battery life of approximately 12 hours.

Participants are instructed to remove and recharge the sensors nightly. Data are continuously recorded and stored locally on the devices, then returned by pre-paid mail after each week of monitoring. Upon receipt, the raw data are uploaded to a secure AWS cloud server, processed using validated algorithms, and results downloaded for analysis.

The algorithms used to calculate the measures of gait and turning were the same for the laboratory and daily life data, as were detailed previously [13,68,69]. In summary, the daily life algorithm first searches for possible bouts of walking from inertial sensor data from the feet using a time-domain approach. Second, individual steps are combined into potential bouts of walking if the duration from one step to the next step is less than 2.5 seconds. Finally, each possible bout that contains at least 3 seconds in duration and at least 3 steps is processed with the same gait algorithms included in Mobility Lab V2 (APDM Wearable Technologies, A Clario company) (20). For the gait measures reported in this paper, we calculated a mean and variability across all strides over the week of recording and included only the periods of straight walking. Straight walking were periods of walking in which the heading angle of the foot during stance changed by no more than 20 degrees during a single stride and that did not contain detected turns as determined from the lumbar sensor [74]. For turning measures, we used a previously published algorithm to detect and characterize each turn [74]. This approach ensured consistency in identifying turns across both structured lab settings and free-living conditions.

### 3.5. Intervention

#### 3.5.1. Device hardware.

**Fig 3:**
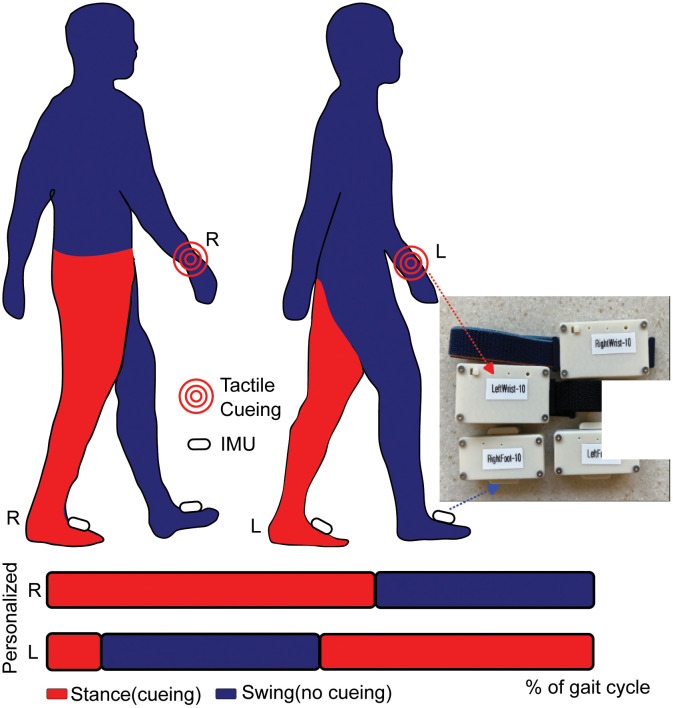
Tactile cueing device. Participants will use 2 IMUs (R and L foot), and 2 vibrotactile units (R and L wrist). In the personalized cueing mode, the wrist receives tactile stimulation when the ipsilateral foot is in the stance phase. The inertial sensors (IMU) identify the stance phase for each foot (red color) during the gait cycle. The picture to the right shows the IMUs and vibrotactile units. In the fixed cueing mode, tactile stimulation will occur at a set frequency based on the person’s gait speed – measured during the laboratory visit. R = Right, L = Left. The IMUs of the cueing device are independent of the IMUs for Home monitoring.

The tactile cueing device was designed and manufactured in our laboratory. An individual device consists of four modules; two are worn on the instep of each foot, and the other two on each wrist (Fig 3). The module worn on the foot contains a microcontroller unit (MCU) with flash storage and an inertial measurement unit (IMU), while the module worn on the wrist contains an MCU and a vibration motor. The wrist and foot modules communicate via Bluetooth Low Energy (BLE). The IMU samples at 104 Hz and the vibration motor is driven at 170 Hz.

All participants will receive this identical sensor hardware, capable of operating in either personalized (closed-loop) or fixed (open-loop) cueing mode based on group allocation (Fig 3).

In the laboratory, the immediate effects of cueing will be evaluated following a 15-minute acclimatization period using fNIRS and wearable sensors.

After Laboratory Visit 1, participants will begin the home monitoring phase. During week 2 of this phase, they will be instructed to use the cueing system for at least 30 minutes per day when they feel in their best ON medication state. Participants will be able to divide the 30 minutes into shorter periods as needed in relation to fatigue, space or other issues. Additionally, we will ask to participants to maintain their medication schedule and refrain from any change in medication or exercise for the duration of the trial.

For both the laboratory visit and home use, the cueing intervention will be stopped if the patients report intolerable discomfort, balance issues, or if they experience an adverse event, such as a fall or other important health problem.

The intensity of the intervention and hardware setup will be the same across both groups.

The personalized cueing (closed-loop) is triggered by the individual’s angular velocity of the foot in real-time and delivers tactile stimulation to the ipsilateral wrist during the stance phase of gait. This modality is personalized and real-time to each step made by the individual (Fig 3).

The fixed cueing (open-loop) delivers tactile stimulation at a frequency matched to the individual’s average gait cycle time, determined during the in-lab portion of their visit using the Mobility Lab V2 algorithms. This value is used to as an input to the vibrotactile device to provide alternating vibration to the left and right wrists that is consistent with the person’s self-selected walking speed. Viewing individual time points of the gait cycle as a percentage of the full duration, vibration will be provided to the left wrist between 5%−45% and vibration to the right wrist will occur between 55%−95%. The vibration will be off outside of these windows, and the pattern will repeat after a gait cycle is completed.

#### 3.5.2. Adherence monitoring.

Participants will receive a printed diary to record the times they put on and removed the sensor each day, along with the timing of their medication and any use of the cueing device. Additionally, when the sensors are returned, we will download data that includes precise timestamps. The cueing device also automatically logs usage times, providing an accurate record of when it was activated.

#### 3.5.3. Stopping rules.

This study will be stopped prior to its completion if: (1) the intervention is associated with adverse effects that call into question the safety of the intervention; (2) difficulty in study recruitment or retention will significantly impact the ability to evaluate the study endpoints; (3) any new information becomes available during the trial that necessitates stopping the trial; or (4) other situations occur that might warrant stopping the trial.

### 3.4. Outcome measures

Primary and secondary outcome measures are summarized in Table 1, which includes the domains and metrics. These measures have been selected based on 1) potential relation to gait automaticity (PFC activity and gait variability) and 2) response to cueing (stride time, LDS, turn duration, turn jerkiness).

Effects of personalized versus fixed tactile cueing during daily life will also be explored via cortical, gait and turning outcomes in Table 1 during week 2 of cueing feasibility at home versus week 1. Daily life gait and turning metrics are exploratory.

Analyses will be performed on an intention to treat basis. However, for the home monitoring, we will exclude participants who will not use the sensors for at least 5 hours per day and 5 days per week. For the cueing intervention, we will exclude participants if they walk with cueing for less than 20 minutes per day or less than 5 days per week.

### 3.5. Statistical methods

#### 3.5.1. Power analysis.

We computed power to detect differences in a larger reduction of PFC activity during personalized, compared to fixed, tactile cueing. Furthermore, we predict that personalized cueing will show less activation of PFC versus fixed cueing (i.e., more automatic). We conservatively assume 54 subjects, 27 for each group (personalized and fixed training), will be included in this analysis, based on recruitment of 60, and allowing for modest dropout (> 10%). We computed power for a repeated-measures ANOVA model with 1 between and 1 within subject effect based on the Geisser-Greenhouse F-test (equivalent to a mixed model). Using patterns of HbO2 levels comparable to previously published results(ref Stuart and Mancini JNPT), assuming between-subject variance of 0.08, modest intra-subject correlation of 0.2, and level of significance at 0.05, we find that power is 82% for the test of between-subject effects (cueing group comparison), and 94% for the test of interaction (differential impact of dual-task versus single-task walking between the personalized- and fixed-cueing groups).

We also computed sample sizes required to achieve at least 80% power for detecting group differences of at least 0.50 SD for measures of walking and turning in daily life with ANCOVA models, conservatively assuming covariate R2 equal to 20%. We set level of significance to 0.02, which is the average alpha level when employing the Holm correction method over 6 outcomes (see below). Under these assumptions, 36 patients (18 per arm) are required to achieve 81.2% power. Thus, our plan to recruit 30 per arm will result in adequately-powered tests while allowing for missing/insufficient data or patient dropout. One of the goals of this trial is to generate preliminary estimates of sensitivity of daily life gait and turning quality due to a cueing intervention. These estimates ultimately will be used to inform power and sample size computations for a larger clinical trial. The small intervention proposed here (30 per group) is not designed to be a highly powered trial itself, but will make use of a randomized, two-group intervention design. All computations were conducted using PASS v15 (PASS 15 Power Analysis and Sample Size Software (2017), NCSS, LLC).

We are not planning interim analysis, and we are not expecting to stop the recruitment early.

#### 3.5.2. Statistical analysis.

The primary outcome will compare PFC and sensory cortex HbO2 while walking and turning between personalized tactile cueing and fixed tactile cueing groups measured during Laboratory Visit 1, without and with cueing. We will average the HbO2 concentrations over 2-minutes of walking (and over 1-minute of 360-degree clockwise and counterclockwise turning), then compare within-subject effects (baseline versus cueing) and between-subject effects (personalized versus fixed cueing) in a repeated measures analysis. Specifically, we will apply mixed-effects models with HbO2 levels (averaged over walking bout) for each person at each assessment time point as the dependent variable. We will include as independent variables: (i) indicator variable for group assignment (personalized or fixed cueing), (ii) indicators for time points (baseline, cueing), and (iii) group by assessment interaction terms. Randomization stratification variables, Hoehn and Yahr, FOG and cognitive status, will be entered into the model as covariates. A random subject intercept will account for correlation between the two repeated measurements within each subject.

Similar linear mixed effects models will be used to demonstrate improvement in walking and turning metrics in the laboratory (carried out with the fNIRS measurements), secondary outcomes. Significance level will be set at 0.05 for testing whether coefficients are different from 0 for primary and secondary outcomes.

We will employ similar linear mixed effects models to investigate whether the two cueing modalities will result in different improvements of walking and turning during daily life. This is an exploratory analysis. For the week of daily mobility without and with cueing, we will summarize walking and turning measures at home (outlined on Table 1) by computing the average over 7 days of daily activity, considering only the recordings with more than 7 hours per day and at least 5 days (Table 1). Randomization stratification variables, Hoehn and Yahr, freezing of gait and cognitive status, will be entered into the model as covariates. Additional covariates would be the life space assessment and activity levels. The effect sizes from models fit for this pilot study will be used to determine the number of subjects required for a future clinical trial.

No Data Monitoring Committee was established for this study, as it was deemed low risk and of limited duration. Safety monitoring will be conducted by the principal investigator and the study team in accordance with the approved protocol

## 4. Discussion

This project aims to characterize the cortical correlates of gait automaticity in PD, evaluate the effects of personalized, versus fixed, tactile cueing on automaticity and explore the feasibility and efficacy of using tactile cueing to improve gait and turning in daily life. The technologies used pose minimal risk and findings could inform more effective, individualized rehabilitation strategies to reduce falls and enhance mobility.

If we find a decrease in PFC activity and an increase in sensory activity only with personalized, closed-loop cueing, and not fixed, open-loop cueing, compared to baseline, this will support our hypothesis that personalized, closed-loop feedback improves gait automaticity. However, we expect to find improvements in walking and turning, measured in the laboratory (reduced stride-time variability, increased gait speed, reduced turning duration and turning jerkiness) with both cueing interventions. We will investigate which cueing modality is superior in improving gait and turning during daily life, when less attention is on gait [75]. If we find a a small effect size, then a greater sample size will be needed for a future, randomized, placebo-controlled clinical trial to improve daily mobility in people with PD.

This trial incorporates several innovative elements. It is among the first to combine portable fNIRS with wearable inertial sensors to assess both cortical activity and gait and turning measures in the laboratory, as well as investigating feasibility of using cueing and measuring effects on gait and turning in the real-world. The use of personalized, closed-loop tactile cueing, which delivers stimulation in real time based on the individual’s gait cycle, represents a major advancement over conventional fixed (open-loop) cueing systems. Unlike auditory or visual cues, the tactile modality targets proprioceptive pathways and may better engage subcortical circuits involved in gait automaticity [38,40].

Another aspect of innovation is the fact that our cueing system is standalone, app-free design, allowing users to benefit from tactile cueing without relying on smartphones or external devices. The tactile stimulus occurs on simple bracelets on the wrists, which are easy to don and provides perceptible vibration to people with PD, who usually cannot detect vibration on the feet [76]. The cueing system has a simple switch to turn the stimulation on or off. This simplicity is especially important for people with Parkinson’s disease, who may experience cognitive or motor challenges that make app navigation difficult. By eliminating the need for digital interfaces, the system may enhance usability, accessibility, and real-world adherence, making it a practical solution for unsupervised use in daily life. The device’s personalized, closed-loop algorithm still operates in real time based on the user’s gait, delivering tailored cues, while maintaining a user-friendly, low-tech interface, which is a novel feature in the field of gait rehabilitation.

Furthermore, using instrumented socks allows for unobtrusive and continuous monitoring of gait in daily life, providing a rich dataset to evaluate how cueing affects mobility outside the laboratory. Likewise, the sensor on the elastic belt around the waist provides accurate measures of turning quality in daily life. By bridging neural, behavioral, and ecological data, this study can help next-generation, personalized rehabilitation strategies in PD and as well as other groups with gait impairments. Gait automaticity, or the ability to multitask while walking, is a critical, functional skill that may be improved with the addition of appropriate rehabilitation, such as cueing.

## Supporting information

S1 File Instructions for fixed and personalized cueing. (DOCX)

S2 File Usability questionnaire for the cueing device. (DOCX)

S3 File CONSORT checklist. (DOCX)

S4 File Notice of Award (NoA), R01, Martina Mancini. (PDF)

## Abbreviations

PD: Parkinson’s disease
fNIRS: functional, near-infrared spectroscopy
EEG: Electroencephalogram
IMU: Inertial Measurement Units
PFC: Premotor cortex
SMA: Supplementary motor area
FoG: Freezing of gait
MDS-UPDRS: Movement Disorders Society Unified Parkinson’s Disease Rating Scale
NFOGQ: New Freezing of Gait Questionnaire
HbO_2_: Cerebral oxy-hemoglobin
HHb: Cerebral deoxy-hemoglobin
PDQ-39: Parkinson’s Disease Quality of Life questionnaire
AS: Apathy Scale
GDS: Geriatric Depression Scale
CLOX 1: Royall’s clock drawing
TMT: Trail-making
MoCA: Montreal Cognitive Assessment (MoCA) [72]
SRT: simple reaction time
CRT: choice reaction time
JLO: judgment of line orientation
AX-CPT: modified attention/vigilance task, the Continuous Performance Test-AX version
LDS: Phase-dependent local dynamic stability.
