# Correction: Personalized versus fixed tactile cueing in Parkinson’s disease: Protocol for a randomized controlled trial on gait automaticity

**DOI:** 10.1371/journal.pone.0341730

**Published:** 2026-01-23

**Authors:** The PLOS One Staff

The first author, Pablo I. Burgos, is incorrectly noted as the corresponding author. The correct corresponding author is Martina Mancini. Martina Mancini’s email address is: mancinim@ohsu.edu.

The publisher apologizes for the error.
