# Supplementary material for: Personalized versus fixed tactile cueing in Parkinson’s disease: Protocol for a randomized controlled trial on gait automaticity
Source: PLoS One. 2025 Nov 21;20(11):e0336859. doi: 10.1371/journal.pone.0336859 (PMC12637899; doi:10.1371/journal.pone.0336859)
Supplement: S1 File — (DOCX) [file pone.0336859.s001.docx]

***Supplementary material***

**SM1. Instructions for fixed and personalized cueing.**

Fixed: “The patterned vibration is programmed to go along with your walking pace, which we assessed during testing. The vibration will alternate between the right and left wrist sensors. The vibration indicates when you need to take a step. When you feel a vibration on your right wrist, pick up your right foot. The same goes for the left side. Do your best to keep in step with the vibration; it’s okay to take some time to adjust as you go. The vibration is meant to assist you in maintaining a consistent walking pace.”

Personalized: “The patterned vibration is programmed to continuously adjust to your current walking pace. The vibration will alternate between the right and left wrist sensors. The vibration indicates when you need to take a step. When you feel a vibration on your right wrist, pick up your right foot. The same goes for the left side. Do your best to keep in step with the vibration; it’s okay to take some time to adjust as you go. The vibration is meant to assist you in maintaining a consistent walking pace.”

1. How do you feel about the noise of the device?

1. How did the device influence your daily living?

1. Outside of this research study, do you think this device would be helpful to you for your daily mobility? If the device were on the market, would you consider purchasing it?
