# Supplementary material for: Personalized versus fixed tactile cueing in Parkinson’s disease: Protocol for a randomized controlled trial on gait automaticity
Source: PLoS One. 2025 Nov 21;20(11):e0336859. doi: 10.1371/journal.pone.0336859 (PMC12637899; doi:10.1371/journal.pone.0336859)
Supplement: S2 File — (DOCX) [file pone.0336859.s002.docx]

***Supplementary material***

**SM2. Usability questionnaire for the cueing device**

**Cueing Device Feedback Questionnaire**

1. Was the device easy to use overall?

1. Was the device easy to wear and/or comfortable?

1. Describe any ease and/or difficulty when taking the device on and off.

1. Was it easy to charge the device?

1. Vibration Strength (scale 0 to 10)

Too Weak Too Strong
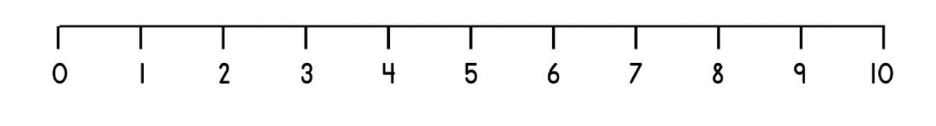


1. How do you feel about the noise of the device?

1. How did the device influence your daily living?

1. Outside of this research study, do you think this device would be helpful to you for your daily mobility? If the device were on the market, would you consider purchasing it?
