# Supplementary material for: Personalized versus fixed tactile cueing in Parkinson’s disease: Protocol for a randomized controlled trial on gait automaticity
Source: PLoS One. 2025 Nov 21;20(11):e0336859. doi: 10.1371/journal.pone.0336859 (PMC12637899; doi:10.1371/journal.pone.0336859)
Supplement: S4 File — (PDF) [file pone.0336859.s004.pdf]

Department of Health and Human Services

National Institutes of Health

EUNICE KENNEDY SHRIVER NATIONAL INSTITUTE OF CHILD HEALTH & HUMAN DEVELOPMENT

Notice of Award

FAIN# R01HD110389

Federal Award Date

02/16/2023

Recipient Information

1. Recipient Name

OREGON HEALTH & SCIENCE UNIVERSITY
3181 SW SAM JACKSON PARK RD

PORTLAND, 97239

2. Congressional District of Recipient

03

3. Payment System Identifier (ID)

1931176109A1

4. Employer Identification Number (EIN)

931176109

5. Data Universal Numbering System (DUNS)

096997515

6. Recipient's Unique Entity Identifier

NPSNT86JKN51

7. Project Director or Principal Investigator

Martina Mancini, ENGD
Assistant Professor
mancinim@ohsu.edu
503-418-2602

8. Authorized Official

Elizabeth S Sobolewski

Federal Agency Information

9. Awarding Agency Contact Information

Saiyda Khan
Scientific Review Specialist
EUNICE KENNEDY SHRIVER NATIONAL INSTITUTE OF CHILD HEALTH & HUMAN DEVELOPMENT
khansa@mail.nih.gov
(301) 496-5001

10. Program Official Contact Information

Susan F. Marden

EUNICE KENNEDY SHRIVER NATIONAL INSTITUTE OF CHILD HEALTH & HUMAN DEVELOPMENT
mardens@mail.nih.gov
301-435-7767

Federal Award Information

11. Award Number

1R01HD110389-01

12. Unique Federal Award Identification Number (FAIN)

R01HD110389

13. Statutory Authority

42 USC 241 42 CFR 52

14. Federal Award Project Title

Cortical correlates of gait in Parkinson's disease: impact of medication and cueing

15. Assistance Listing Number

93.865

16. Assistance Listing Program Title

Child Health and Human Development Extramural Research

17. Award Action Type

New Competing

18. Is the Award R&D?

Yes

Summary Federal Award Financial Information

**19. Budget Period Start Date** 02/23/2023 **– End Date** 01/31/2024	
**20. Total Amount of Federal Funds Obligated by this Action**	$577,850
20 a. Direct Cost Amount	$457,853
20 b. Indirect Cost Amount	$119,997
**21.** Authorized Carryover	
**22.** Offset	
**23.** Total Amount of Federal Funds Obligated this budget period	$577,850
**24. Total Approved Cost Sharing or Matching, where applicable**	$0
**25. Total Federal and Non-Federal Approved this Budget Period**	$577,850
**26. Project Period Start Date** 02/23/2023 **– End Date** 01/31/2028	
**27.** Total Amount of the Federal Award including Approved Cost Sharing or Matching this Project Period	$577,850

28. Authorized Treatment of Program Income

Additional Costs

29. Grants Management Officer - Signature

Saiyda Khan

30. Remarks

Acceptance of this award, including the "Terms and Conditions," is acknowledged by the recipient when funds are drawn down or otherwise

requested from the grant payment system.

SECTION I – AWARD DATA – 1R01HD110389-01

Principal Investigator(s):
Martina Mancini, ENGD

Award e-mailed to: orserv@ohsu.edu

Dear Authorized Official:

The National Institutes of Health hereby awards a grant in the amount of $577,850 (see "Award Calculation" in Section I and "Terms and Conditions" in Section III) to OREGON HEALTH & SCIENCE UNIVERSITY in support of the above referenced project. This award is pursuant to the authority of 42 USC 241 42 CFR 52 and is subject to the requirements of this statute and regulation and of other referenced, incorporated or attached terms and conditions.

Acceptance of this award, including the "Terms and Conditions," is acknowledged by the recipient when funds are drawn down or otherwise requested from the grant payment system.

Each publication, press release, or other document about research supported by an NIH award must include an acknowledgment of NIH award support and a disclaimer such as "Research reported in this publication was supported by the Eunice Kennedy Shriver National Institute Of Child Health & Human Development of the National Institutes of Health under Award Number R01HD110389. The content is solely the responsibility of the authors and does not necessarily represent the official views of the National Institutes of Health." Prior to issuing a press release concerning the outcome of this research, please notify the NIH awarding IC in advance to allow for coordination.

Award recipients must promote objectivity in research by establishing standards that provide a reasonable expectation that the design, conduct and reporting of research funded under NIH awards will be free from bias resulting from an Investigator's Financial Conflict of Interest (FCOI), in accordance with the 2011 revised regulation at 42 CFR Part 50 Subpart F. The Institution shall submit all FCOI reports to the NIH through the eRA Commons FCOI Module. The regulation does not apply to Phase I Small Business Innovative Research (SBIR) and Small Business Technology Transfer (STTR) awards. Consult the NIH website http://grants.nih.gov/grants/policy/coi/ for a link to the regulation and additional important information.

If you have any questions about this award, please direct questions to the Federal Agency contacts.

Sincerely yours,

Saiyda Khan
Grants Management Officer
EUNICE KENNEDY SHRIVER NATIONAL INSTITUTE OF CHILD HEALTH & HUMAN DEVELOPMENT

Additional information follows

Cumulative Award Calculations for this Budget Period (U.S. Dollars)

**Salaries and Wages**	$253,228
**Fringe Benefits**	$82,657
**Personnel Costs (Subtotal)**	$335,885
**Equipment**	$34,400
**Materials & Supplies**	$3,440
**Travel**	$2,580
**Other**	$5,504
**Subawards/Consortium/Contractual Costs**	$73,464
**Publication Costs**	$2,580

**Federal Direct Costs**	$457,853
**Federal F&A Costs**	$119,997
**Approved Budget**	$577,850
**Total Amount of Federal Funds Authorized (Federal Share)**	$577,850
**TOTAL FEDERAL AWARD AMOUNT**	$577,850
**AMOUNT OF THIS ACTION (FEDERAL SHARE)**	$577,850

**SUMMARY TOTALS FOR ALL YEARS** (for this Document Number)		
**YR**	**THIS AWARD**	**CUMULATIVE TOTALS**
1	$577,850	$577,850
2	$552,801	$552,801
3	$552,801	$552,801
4	$531,958	$531,958
5	$528,644	$528,644

Recommended future year total cost support, subject to the availability of funds and satisfactory progress of the project

Fiscal Information:

Payment System Identifier: 1931176109A1
Document Number: RHD110389A
PMS Account Type: P (Subaccount)
Fiscal Year: 2023

IC	CAN	2023	2024	2025	2026	2027
HD	8471483	$577,850	$552,801	$552,801	$531,958	$528,644

Recommended future year total cost support, subject to the availability of funds and satisfactory progress of the project

NIH Administrative Data:
PCC: NCMRR-SM / **OC**: 41021 / **Released**: Khan, Saiyda 02/16/2023
Award Processed: 02/17/2023 12:07:50 AM

SECTION II – PAYMENT/HOTLINE INFORMATION – 1R01HD110389-01

For payment and HHS Office of Inspector General Hotline information, see the NIH Home Page at http://grants.nih.gov/grants/policy/awardconditions.htm

SECTION III – STANDARD TERMS AND CONDITIONS – 1R01HD110389-01

This award is based on the application submitted to, and as approved by, NIH on the above-titled project and is subject to the terms and conditions incorporated either directly or by reference in the following:

a. The grant program legislation and program regulation cited in this Notice of Award.
b. Conditions on activities and expenditure of funds in other statutory requirements, such as those included in appropriations acts.
c. 45 CFR Part 75.
d. National Policy Requirements and all other requirements described in the NIH Grants Policy Statement, including addenda in effect as of the beginning date of the budget period.
e. Federal Award Performance Goals: As required by the periodic report in the RPPR or in the final progress report when applicable.
f. This award notice, INCLUDING THE TERMS AND CONDITIONS CITED BELOW.

(See NIH Home Page at http://grants.nih.gov/grants/policy/awardconditions.htm for certain references cited above.)

Research and Development (R&D): All awards issued by the National Institutes of Health (NIH) meet the definition of "Research and Development" at 45 CFR Part§ 75.2**.** As such, auditees should identify NIH awards as part of the R&D cluster on the Schedule of Expenditures of Federal Awards (SEFA). The auditor should test NIH awards for compliance as instructed in Part V, Clusters of Programs. NIH recognizes that some awards may have another classification for purposes of indirect costs. The auditor is not required to report the disconnect (i.e., the award is classified as R&D for Federal Audit Requirement purposes but non-research for indirect cost rate purposes), unless the auditee is charging indirect costs at a rate other than the rate(s) specified in the award document(s).

This institution is a signatory to the Federal Demonstration Partnership (FDP) Phase VII Agreement which requires active institutional participation in new or ongoing FDP demonstrations and pilots.

An unobligated balance may be carried over into the next budget period without Grants Management Officer prior approval.

This grant is subject to Streamlined Noncompeting Award Procedures (SNAP).

This award is subject to the requirements of 2 CFR Part 25 for institutions to obtain a unique entity identifier (UEI) and maintain an active registration in the System for Award Management (SAM). Should a consortium/subaward be issued under this award, a UEI requirement must be included. See http://grants.nih.gov/grants/policy/awardconditions.htm for the full NIH award term implementing this requirement and other additional information.

This award has been assigned the Federal Award Identification Number (FAIN) R01HD110389. Recipients must document the assigned FAIN on each consortium/subaward issued under this award.

Based on the project period start date of this project, this award is likely subject to the Transparency Act subaward and executive compensation reporting requirement of 2 CFR Part 170. There are conditions that may exclude this award; see http://grants.nih.gov/grants/policy/awardconditions.htm for additional award applicability information.

In accordance with P.L. 110-161, compliance with the NIH Public Access Policy is now mandatory. For more information, see NOT-OD-08-033 and the Public Access website: http://publicaccess.nih.gov/.

This award provides support for one or more clinical trials. By law (Title VIII, Section 801 of Public Law 110-85), the "responsible party" must register "applicable clinical trials" on the ClinicalTrials.gov Protocol Registration System Information Website. NIH encourages registration of all trials whether required under the law or not. For more information, see http://grants.nih.gov/ClinicalTrials_fdaaa/

Recipients must administer the project in compliance with federal civil rights laws that prohibit discrimination on the basis of race, color, national origin, disability, age, and comply with applicable conscience protections. The recipient will comply with applicable laws that prohibit discrimination on the basis of sex, which includes discrimination on the basis of gender identity, sexual orientation, and pregnancy. Compliance with these laws requires taking reasonable steps to provide meaningful access to persons with limited English proficiency and providing programs that are accessible to and usable by persons with disabilities. The HHS Office for Civil Rights provides guidance on complying with civil rights laws enforced by HHS. See https://www.hhs.gov/civil-rights/for-providers/provider-obligations/index.html and https://www.hhs.gov/.

- Recipients of FFA must ensure that their programs are accessible to persons with limited English proficiency. For guidance on meeting the legal obligation to take reasonable steps to ensure meaningful access to programs or activities by limited English proficient individuals, see https://www.hhs.gov/civil-rights/for-individuals/special-topics/limited-english-proficiency/fact-sheet-guidance/index.html and https://www.lep.gov.
- For information on an institution's specific legal obligations for serving qualified individuals with disabilities, including providing program access, reasonable modifications, and to provide effective communication, see http://www.hhs.gov/ocr/civilrights/understanding/disability/index.html.
- HHS funded health and education programs must be administered in an environment free of sexual harassment; see https://www.hhs.gov/civil-rights/for-individuals/sex-discrimination/index.html. For information about NIH's commitment to supporting a safe and respectful work environment, who to contact with questions or concerns, and what NIH's expectations are for institutions and the individuals supported on NIH-funded awards, please see https://grants.nih.gov/grants/policy/harassment.htm.
- For guidance on administering programs in compliance with applicable federal religious nondiscrimination laws and applicable federal conscience protection and associated anti-discrimination laws, see https://www.hhs.gov/conscience/conscience-protections/index.html and https://www.hhs.gov/conscience/religious-freedom/index.html.

In accordance with the regulatory requirements provided at 45 CFR 75.113 and Appendix XII to 45 CFR Part 75, recipients that have currently active Federal grants, cooperative agreements, and procurement contracts with cumulative total value greater than $10,000,000 must report and maintain information in the System for Award Management (SAM) about civil, criminal, and administrative proceedings in connection with the award or performance of a Federal award that reached final disposition within the most recent five-year period. The recipient must also make semiannual disclosures regarding such proceedings. Proceedings information will be made publicly available in the designated integrity and performance system (currently the Federal Awardee Performance and Integrity Information System (FAPIIS)). Full reporting requirements and procedures are found in Appendix XII to 45 CFR Part 75. This term does not apply to NIH fellowships.

Treatment of Program Income:
Additional Costs

SECTION IV – HD SPECIFIC AWARD CONDITIONS – 1R01HD110389-01

Clinical Trial Indicator: Yes
This award supports one or more NIH-defined Clinical Trials. See the NIH Grants Policy Statement Section 1.2 for NIH definition of Clinical Trial.

Due to the impact of the Coronavirus disease 2019 (COVID-19) outbreak, NICHD will consider providing greater flexibilities to recipients in meeting administrative, financial management and audit requirements. Please contact the Grants Management Specialist and Program Official indicated on this Notice of Award for more information.

****

In order to meet NICHD program objectives within FY2023 budget constraints, this grant is reduced **14** percent below the level recommended by the Integrated Review Group. Future year levels of support are determined by applying the same administrative reduction.

****
This award includes funds for twelve months of support but is awarded for less than twelve months. Noncompeting Continuation awards will cycle on **February 1.**

****

This award is subject to the requirements indicated in **PA20-183**, which are hereby incorporated by reference. See the NIH Funding Opportunity Announcements site.

****
In accordance with the NICHD FY2023 fiscal policy, escalation on recurring costs has been removed. See NICHD Funding Strategies for Fiscal Year 2023.

****
No award funds shall be used to pay the salary of an individual at a rate in excess of the current salary cap. This award has been adjusted accordingly See NIH Guide Notice NOT-OD-23-056.

****

For all competing applications or new protocols, the NICHD expects investigators for ALL NICHD Clinical Trials to abide by the requirements stated in NIH Guide Notice NOT-HD-20-036 "NICHD Data Safety Monitoring Guidelines for Extramural Clinical Trials and Clinical Research". All NICHD applications which include Clinical Trials must include a Data Safety Monitoring Plan. All NIH-sponsored multi-site clinical trials, NIH-defined Phase III clinical trials and some single site clinical trials that pose potential risk to participants require Data and Safety Monitoring Board (DSMB) oversight. Applicants are expected to establish an independent, external DSMB when required by this policy.

For all competing applications or new protocols, the NICHD expects investigators for ALL human subject research to abide by the requirements stated in NIH Guide Notice NOT-HD-20-035 "NICHD Serious Adverse Event, Unanticipated Problem, and Serious Adverse Event Reporting Guidance".

****
The clinical trial(s) supported by this award are subject to the Dissemination Plan specified in the **application** dated **02/22/2022** and the NIH policy on Dissemination of NIH-Funded Clinical Trial Information. The policy states that the clinical trial(s) funded by this award will be registered in ClinicalTrials.gov not later than 21 calendar days after enrollment of the first participant and that primary summary results will be reported in ClinicalTrials.gov not later than one year after the trial completion date. The reporting of summary results is required even if the primary trial completion date occurs after the period of performance.

This award is subject to additional certification requirements with submission of the Annual, Interim and Final Research Performance Progress Reports (RPPR). The recipient must agree to the following annual certification when submitting each RPPR. By submitting the RPPR, the Signing Official (SO) signifies compliance, as follows:

In submitting this RPPR, the SO (or PD/PI with delegated authority), certifies to the best of their knowledge that, for all clinical trials funded under this NIH award, the recipient and all investigators conducting NIH-funded clinical trials comply with the recipient's plan addressing compliance with the Dissemination of NIH-Funded Clinical Trial Information policy. Any clinical trial funded in whole or in part under this award has been registered in ClinicalTrials.gov or will be registered not later than 21 calendar days after enrollment of the first participant. Summary

results have been submitted to [ClinicalTrials.gov] or will be submitted not later than one year after the trial completion date, even if the trial completion date occurs after the period of performance.

****

Clinical Trial Study/Studies:
407908

This Clinical Trial Study or Studies listed above have been determined by NICHD to be considered **LOW** risk. Oversight by NICHD will occur in the standard manner through the annual RPPR. An annual update (no additional reports) on the status of the milestones included in Section 6 of the eRA HSS and any additional agreed-upon milestones are due in the RPPR. Information and procedures concerning these requirements are available on the NICHD Policies on Clinical Research site.

SPREADSHEET SUMMARY
AWARD NUMBER: 1R01HD110389-01

INSTITUTION: OREGON HEALTH & SCIENCE UNIVERSITY

Budget	Year 1	Year 2	Year 3	Year 4	Year 5
Salaries and Wages	$253,228	$261,798	$261,798	$251,064	$251,064
Fringe Benefits	$82,657	$84,963	$84,963	$79,881	$79,881
Personnel Costs (Subtotal)	$335,885	$346,761	$346,761	$330,945	$330,945
Equipment	$34,400				
Materials & Supplies	$3,440	$3,440	$3,440	$3,440	$3,440
Travel	$2,580	$2,580	$2,580	$2,580	$2,580
Other	$5,504	$7,774	$7,774	$7,800	$5,289
Subawards/Consortium/Contractual Costs	$73,464	$73,463	$73,463	$73,463	$73,463
Publication Costs	$2,580	$2,580	$2,580	$2,580	$2,580
TOTAL FEDERAL DC	$457,853	$436,598	$436,598	$420,808	$418,297
TOTAL FEDERAL F&A	$119,997	$116,203	$116,203	$111,150	$110,347
TOTAL COST	$577,850	$552,801	$552,801	$531,958	$528,644

Facilities and Administrative Costs	Year 1	Year 2	Year 3	Year 4	Year 5
F&A Cost Rate 1	32%	32%	32%	32%	32%
F&A Cost Base 1	$374,990	$363,135	$363,135	$347,345	$344,834
F&A Costs 1	$119,997	$116,203	$116,203	$111,150	$110,347
